# An Ontology-Based Approach to Improving Medication Appropriateness in Older Patients: Algorithm Development and Validation Study

**DOI:** 10.2196/45850

**Published:** 2023-07-10

**Authors:** Elena Calvo-Cidoncha, Julián Verdinelli, Javier González-Bueno, Alfonso López-Soto, Concepción Camacho Hernando, Xavier Pastor-Duran, Carles Codina-Jané, Raimundo Lozano-Rubí

**Affiliations:** 1 Pharmacy Service, Hospital Clínic of Barcelona, Barcelona, Spain; 2 Clinical Informatics Service, Hospital Clínic of Barcelona, Barcelona, Spain; 3 Pharmacy Service, Hospital Dos de Maig, Consorci Sanitari Integral, Barcelona, Spain; 4 Geriatric Unit, Department of Internal Medicine, Hospital Clínic of Barcelona, Barcelona, Spain

**Keywords:** biological ontologies, decision support systems, inappropriate prescribing, elderly, medication regimen complexity, anticholinergic drug burden, trigger tool, clinical, ontologies, pharmacy, medication, decision support, pharmaceutic, pharmacology, chronic condition, chronic disease, domain, adverse event, ontology-based, alert

## Abstract

**Background:**

Inappropriate medication in older patients with multimorbidity results in a greater risk of adverse drug events. Clinical decision support systems (CDSSs) are intended to improve medication appropriateness. One approach to improving CDSSs is to use ontologies instead of relational databases. Previously, we developed OntoPharma—an ontology-based CDSS for reducing medication prescribing errors.

**Objective:**

The primary aim was to model a domain for improving medication appropriateness in older patients (chronic patient domain). The secondary aim was to implement the version of OntoPharma containing the chronic patient domain in a hospital setting.

**Methods:**

A 4-step process was proposed. The first step was defining the domain scope. The chronic patient domain focused on improving medication appropriateness in older patients. A group of experts selected the following three use cases: medication regimen complexity, anticholinergic and sedative drug burden, and the presence of triggers for identifying possible adverse events. The second step was domain model representation. The implementation was conducted by medical informatics specialists and clinical pharmacists using Protégé-OWL (Stanford Center for Biomedical Informatics Research). The third step was OntoPharma-driven alert module adaptation. We reused the existing framework based on SPARQL to query ontologies. The fourth step was implementing the version of OntoPharma containing the chronic patient domain in a hospital setting. Alerts generated from July to September 2022 were analyzed.

**Results:**

We proposed 6 new classes and 5 new properties, introducing the necessary changes in the ontologies previously created. An alert is shown if the Medication Regimen Complexity Index is ≥40, if the Drug Burden Index is ≥1, or if there is a trigger based on an abnormal laboratory value. A total of 364 alerts were generated for 107 patients; 154 (42.3%) alerts were accepted.

**Conclusions:**

We proposed an ontology-based approach to provide support for improving medication appropriateness in older patients with multimorbidity in a scalable, sustainable, and reusable way. The chronic patient domain was built based on our previous research, reusing the existing framework. OntoPharma has been implemented in clinical practice and generates alerts, considering the following use cases: medication regimen complexity, anticholinergic and sedative drug burden, and the presence of triggers for identifying possible adverse events.

## Introduction

Medical advances have resulted in a rise of life expectancy. The prevalence of multimorbidity, which is defined as the coexistence of 2 or more chronic conditions, tends to be higher among older people [1]. As a result, the use of multiple medicines, which is commonly referred to as *polypharmacy*, has become a common phenomenon in this population [2,3].

Polypharmacy increases the risk of inappropriate medication [4,5], leading to a greater risk of adverse drug events (ADEs) [6]. ADEs are associated with hospital admissions, higher mortality rates, and increased health care expenditures [7-10]; therefore, improving medication appropriateness in older patients with multimorbidity is a priority [11].

One approach to improving medication appropriateness is to use clinical decision support systems (CDSSs) for assistance during the prescription process. CDSSs are intended to improve health care delivery by enhancing medical decisions with targeted clinical knowledge and patient information [12,13]. Relational databases are the predominant choice when it comes to designing a CDSS. However, due to the main challenges of CDSSs, such as the lack of interoperability or alert fatigue [14-16], there is increasing interest in using ontology-based CDSSs to overcome these challenges. An ontology is an explicit conceptualization of the entities of a domain [17,18]. Because ontologies add semantics to models, they enhance the reusability of data and are more efficient in dealing with changing requirements and maintenance requirements [19-21].

Previously, we used Protégé-OWL (Stanford Center for Biomedical Informatics Research) to develop OntoPharma—an ontology-based CDSS for reducing medication prescribing errors [22]. The domains addressed by OntoPharma include the identification and technical data of medicinal products, as well as data on drug appropriateness for ensuring the safe use of medicines. These domains were addressed in the following four use cases: maximum dosage alerts, a drug-drug interaction checker, renal failure adjustment, and a drug allergy checker. OntoPharma is currently implemented in a tertiary referral hospital.

Alerts generated by OntoPharma are, nowadays, commonly available. To leverage the ease of using ontologies to represent rich and complex knowledge, the modeling of drug knowledge that is absent in usual commercial databases is needed.

For this reason and on the basis of our previous research, the primary aim of this study was to model a domain for improving medication appropriateness in older patients with multimorbidity (hereinafter called the *chronic patient domain*). The secondary aim was to implement the version of OntoPharma containing the chronic patient domain in a hospital setting.

## Methods

This study was conducted between 2020 and 2022 at a 710-bed tertiary hospital in Spain, which was equipped with computerized physician order entry (CPOE) and an electronic health record (EHR) system provided by SAP SE. The following 4-step development process was designed: defining the domain scope, representing the domain in the model, adapting the OntoPharma-driven alert module, and implementing the version of OntoPharma containing the chronic patient domain in a hospital setting.

### Ethics Approval

This study was approved by the ethics committee of the Hospital Clínic of Barcelona (reference number HCB/2019/0735).

### Defining the Domain Scope

#### Overview of the Domain Scope

The chronic patient domain focused on improving medication appropriateness in older patients with multimorbidity. Given the dimension of the domain, we decided to establish an expert advisory panel to limit the scope of the domain. The group of experts included geriatricians and clinical pharmacists, of whom all were members of the C3RG (Central Catalonia Chronicity Research Group) and had expertise in ensuring medication appropriateness in older patients with multimorbidity. Focus group sessions yielded consensus on the importance of the following three use cases: medication regimen complexity, anticholinergic and sedative drug burden, and the presence of triggers for identifying possible adverse events.

#### Medication Regimen Complexity

Complex medication regimens are challenging for patients, which may impact medication adherence and safety [23,24]. The Medication Regimen Complexity Index (MRCI), which was developed by George et al [25], is currently the most widely used scale for assessing medication regimen complexity. Medication complexity considers more factors than a simple medication count. The MRCI consists of 65 items, including weighted scores for types of prescribed dosage forms (section A), dosing frequency (section B), and additional administration instructions (section C). The sum of the scores of the three sections provides a total score, with higher scores indicating greater regimen complexity.

The MRCI has been translated and validated for other languages, including Spanish (Spanish MRCI [MRCI-E]) [26]. We used the MRCI-E as a source of information.

Section A provides weights for 32 dosage form and administration route combinations. For example, an oral tablet medication is given a weight of 1. More complex combinations result in higher weights.

Section B provides weights for 23 dosing frequencies (“scheduled” or “as needed”). The “once daily” frequency is used as the baseline (weight of 1), on which the other weightings are built.

Section C provides weights for 10 additional instructions that a patient may need to follow in adhering to a prescribed regimen. Additional administration instructions are related to taking medication at specific times, taking medication in relation to food, taking multiple units at one time, and needing to break or crush a tablet or needing to taper or increase a dose.

#### Anticholinergic and Sedative Drug Burden

*Anticholinergic burden* is defined as the cumulative effect of taking 1 or more drugs that are capable of causing anticholinergic adverse effects, and the load increases with the number of medications prescribed [27]. Anticholinergic toxicity is a common problem in older people. Anticholinergic effects are associated with peripheral manifestations (urinary retention, constipation, decreased secretions, etc) and central manifestations (delirium, cognitive disorders, and functional disorders) [27,28].

Several tools have been developed to estimate anticholinergic burden by giving a score to drugs according to their anticholinergic potential [29]. The Drug Burden Index (DBI) is the only scale that accounts for a patient’s dose [30]. In addition, the DBI considers not only anticholinergic effects but also sedative effects. The total DBI exposure is calculated as the sum of exposure to any DBI medication, according to the following formula:


(1) DBI=∑D/(δ+D)

where “D” is the daily dose taken and “δ” is the minimum effective daily dose for that drug.

Byrne et al [31] provided a master DBI list containing a final list of DBI medications and their minimum effective daily doses. The master DBI list included 156 entries. Each entry consisted of the following fields: drug description (ingredient), World Health Organization Anatomical Therapeutic Classification codes, anticholinergic and sedative effects, and minimum effective daily dose (expressed as mg) by route of administration (parenteral, sublingual, buccal, transdermal, rectal, and inhalation).

#### Triggers

A trigger is defined as a flag, occurrence, or prompt that alerts reviewers to initiate further in-depth investigations regarding a patient’s record to determine the presence or absence of an adverse event [32]. An example of a trigger is a potassium level of <2.9 mEq/L in a patient with loop diuretics. Triggers are based on the assumption that any new condition may be due to the use of a drug. Multiple sets of triggers have been developed. Guzmán et al [33] identified the most appropriate triggers for detecting ADEs in older patients with multiple chronic conditions.

The trigger set developed by Guzmán et al [33] included a total of 51 entries. Each entry consisted of the following fields: high-alert medications for patients with chronic illnesses (therapeutic class or ingredient) and triggers for detecting potential ADEs (11 care module triggers, 9 antidote- and treatment-based triggers, 11 medication concentration–based triggers, 18 triggers based on abnormal laboratory values, and 1 emergency department trigger).

### Domain Model Representation

Data sets were not organized in a predefined format. Prior to modeling the chronic patient domain through ontologies, we processed all of the information in a relational database to clean the data, detect redundancies, and detect relationships between different concepts.

To add this new domain to OntoPharma, we built on our previous research by using the existing framework, which was composed of 3 ontologies (*Drugs*, *Decision support system* [*DSS*], and *Local pharmacy*) [22]. The *Drugs* ontology was designed to represent the identification and technical data of medicinal products. The *DSS* ontology provides data on drug appropriateness. The *Local pharmacy* ontology was designed to represent local concepts from EHRs and CPOE in order to ensure interoperability.

The design, development, and maintenance of the chronic patient domain was driven by medical informatics specialists and clinical pharmacists. The information was represented in the Web Ontology Language (OWL) [34]. For encoding the OWL ontologies, we used the Protégé 3.5 editor tool [35]. The concepts of the chronic patient domain were organized hierarchically, following a top-down approach, as we did with all previous domains of OntoPharma. The development of the class hierarchy, the defining of properties, and the slotting of concepts were carried out at the same time. Finally, we defined individual instances of the classes represented.

### OntoPharma-Driven Alert Module Adaptation

We reused the OntoPharma-driven alert module that was proposed in our previous research [22]. The integration between the CPOE system and the ontologies was performed through a REST API. A REST API call was published (in JSON format) each time a clinician added a new medication in the CPOE system, modified an existing one, or requested on-demand CDSS information. The request contained patient-specific clinical data. SPARQL (Apache Jena Fuseki server) was used to query ontologies [36]. After applying the queries, a returning REST API, with the results, was published.

It was necessary to update the content of the REST API published each time OntoPharma was triggered. We specifically had to add more laboratory parameters (to date, the only one considered was glomerular filtration rate). New local concepts were manually mapped with existing concepts in the ontologies. In addition, we created new SPARQL queries to ensure the safe use of medicines in older patients.

Alerts were shown in the CPOE system in cases of high medication regimen complexity, in cases of high anticholinergic and sedative drug burden, or in cases where triggers for detecting ADEs in older patients were present. In addition, patients were required to be older than 65 years.

In accordance with the recommendations of end users, the user interface proposed in the previous paper [22] was slightly modified to ensure usability and minimal interference with the clinician’s workflow.

Formal testing was performed to demonstrate that the new version of the ontology-driven alert module met functional requirements. In addition, clinical pharmacists performed manual testing in a control environment (the SAP quality assurance server) to evaluate whether the alert module functioned properly when generating the prescribing alerts.

### Implementation of the Version of OntoPharma Containing the Chronic Patient Domain in a Hospital Setting

In July 2022, the version of OntoPharma containing the chronic patient domain was implemented at one ward of the internal medicine unit, which had capacity for 20 admissions. Informatics staff and clinical pharmacists were responsible for the diffusion and for providing support.

A retrospective analysis of the alerts generated was performed. We included patients who were admitted to the internal medicine ward from July to September 2022. The following patient data were collected: gender, age, duration of hospital stay, and number of medications during hospital stay. We further examined the alerts, including the number of alerts, the types of alerts, clinical relevance, and the acceptance rates.

Quantitative variables were expressed as means and SDs for variables with a normal distribution or as medians and IQRs for variables with a skewed distribution. Qualitative variables were expressed as percentages. Data analysis was carried out by using SPSS 20.0 (IBM Corp).

## Results

### Knowledge Representation Using Ontologies

#### Overview of Ontologies

**Figure 1.**
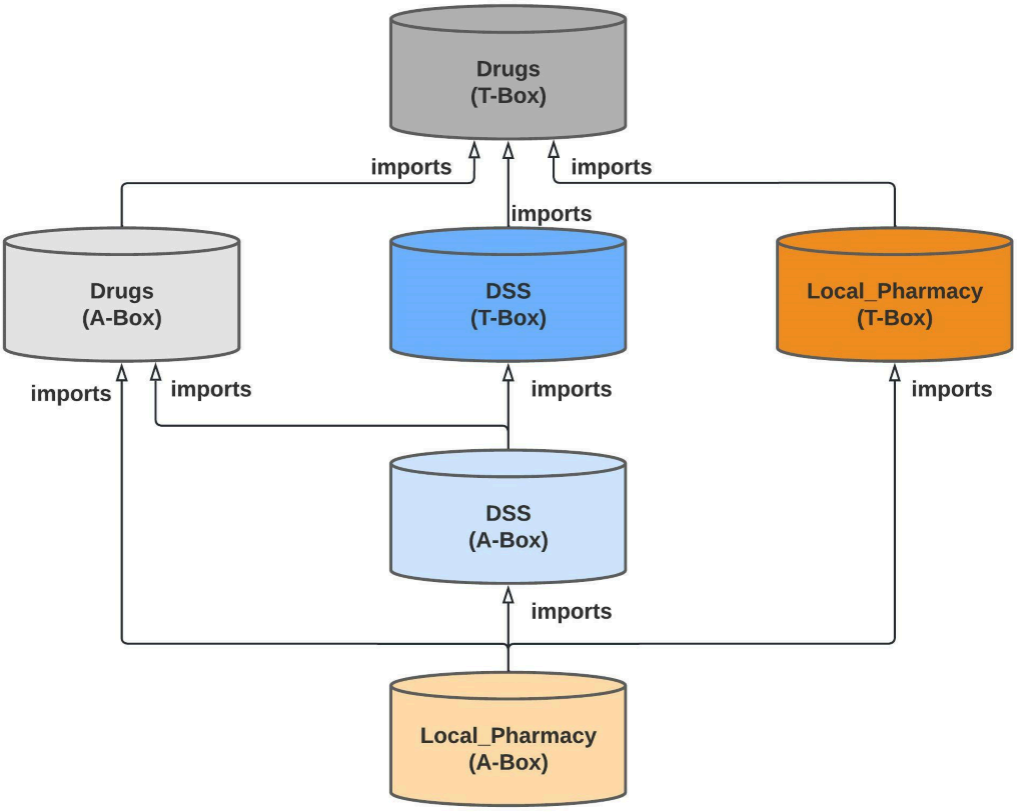
Import schema of the ontologies used in OntoPharma. For modeling drug-related knowledge, 3 ontologies have been developed (*Drugs*, *DSS*, and *Local pharmacy*). Each ontology has been divided into 2 parts. The first part provides concepts and classes (also known as *T-Box*), and the second provides the instances of these concepts (also known as *A-Box*). The three ontologies are interconnected, as shown in Figure 1. DSS: Decision support system.

For modeling the chronic patient domain, we proposed new classes and properties, introducing the necessary changes in the ontologies previously created (*Drugs*, *DSS*, and *Local pharmacy*). The three ontologies are interconnected. The import schema of ontologies is shown in Figure 1.

**Figure 2.**
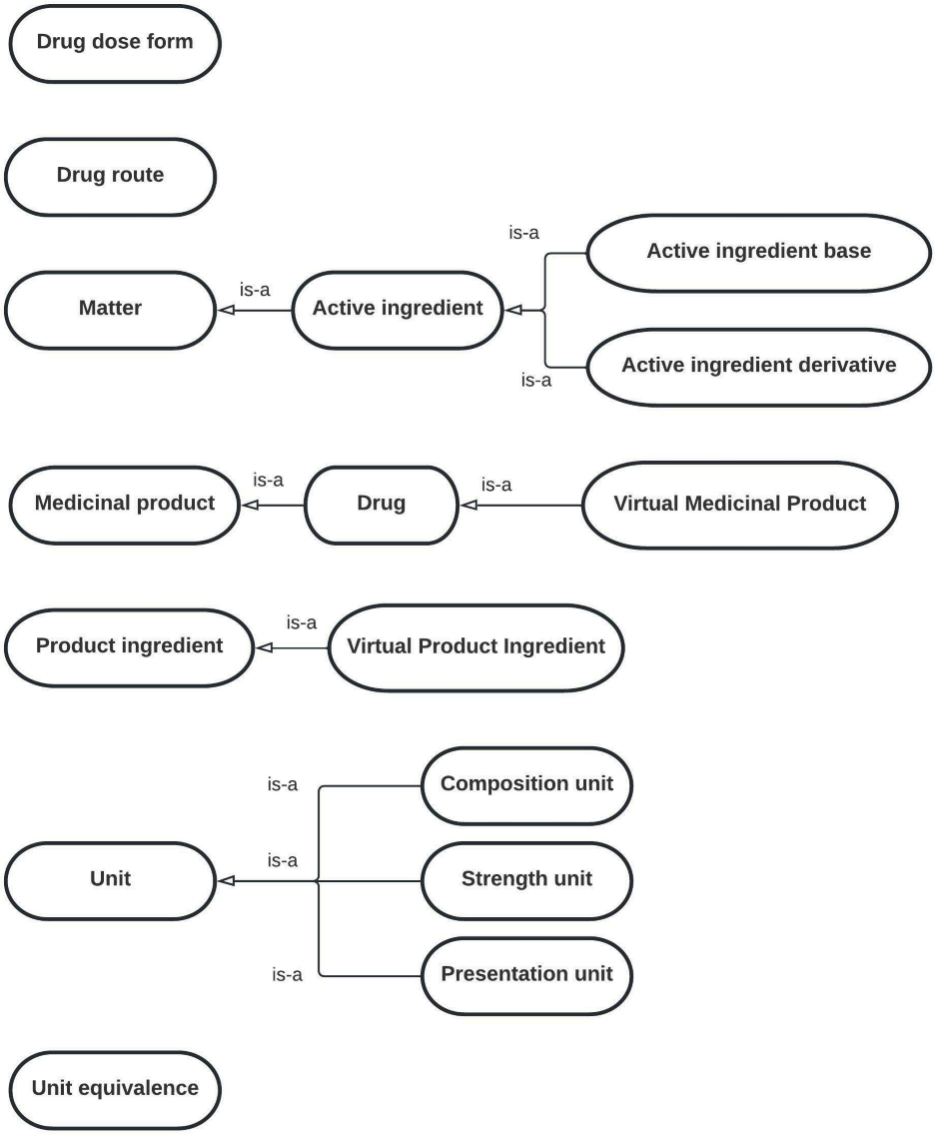
Diagram showing the relationships between classes in the *Drugs* ontology for defining the chronic patient domain. The *Drugs* ontology was designed to represent the identification and technical data of medicinal products.

**Figure 3.**
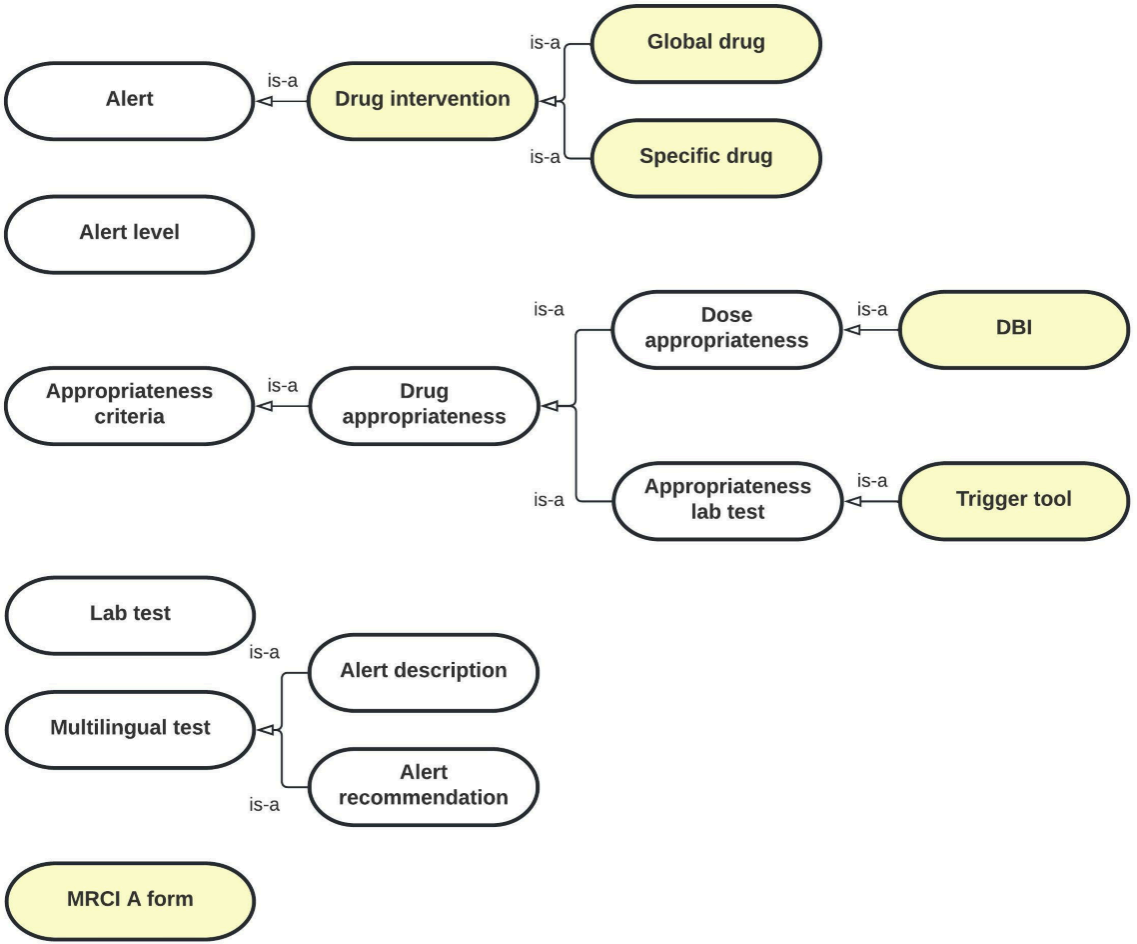
Diagram showing the relationships between classes in the *DSS* ontology for defining the chronic patient domain. The DSS ontology provides data on drug appropriateness. Concepts that were specifically created to define the chronic patient domain are highlighted in yellow. DBI: Drug Burden Index; DSS: Decision support system; MRCI: Medication Regimen Complexity Index.

**Figure 4.**
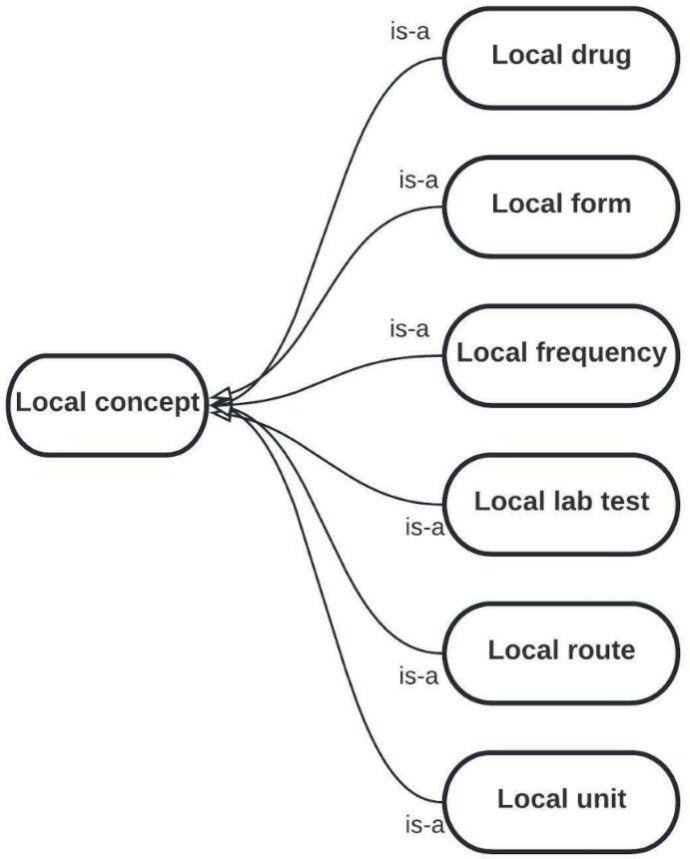
Diagram showing the relationships between classes in the *Local pharmacy* ontology for defining the chronic patient domain. The *Local pharmacy* ontology was designed to represent local concepts from electronic health records and computerized physician order entry. Each local concept is mapped to the corresponding OntoPharma concept.

Figures 2-4 provide diagrams showing the relationships between classes for defining the chronic patient domain in the *Drugs* ontology, *DSS* ontology, and *Local pharmacy* ontology, respectively.

Multimedia Appendix 1 contains a list of the medication knowledge concepts and their definitions, which were used to define the chronic patient domain. Multimedia Appendix 2 contains a list of properties and their facets, which were also used to define the chronic patient domain.

#### Medication Regimen Complexity

The MRCI quantifies drug regimen complexity based on dosage form, dosage frequency, and additional instructions.

To represent the weighted scores for types of prescribed dosage forms (section A), we first created the concept “MRCI A form” (*DSS* ontology), which comprises 30 subclasses for identifying the possible dosage form and route of administration combinations. To provide the weight for each dosage form and route of administration combination, we introduced the property “mrci A weight.”

To represent the weighted scores for dosage frequency (section B), we introduced the following two attached properties within the class “Local frequency” (*Local pharmacy* ontology): “mrci B,” which provides weights for “scheduled” dosing frequencies, and “mrci B PRN,” which provides weights for “as needed” dosing frequencies.

We identified 231 distinct frequency combinations. Frequency weights were assigned, considering that frequency data also contained indicators that qualify for component C scoring, such as indicators to take medication less often than once per day (eg, once every 48 hours) or indicators to take medication at specific times (before a meal, at bedtime, etc).

**Figure 5.**
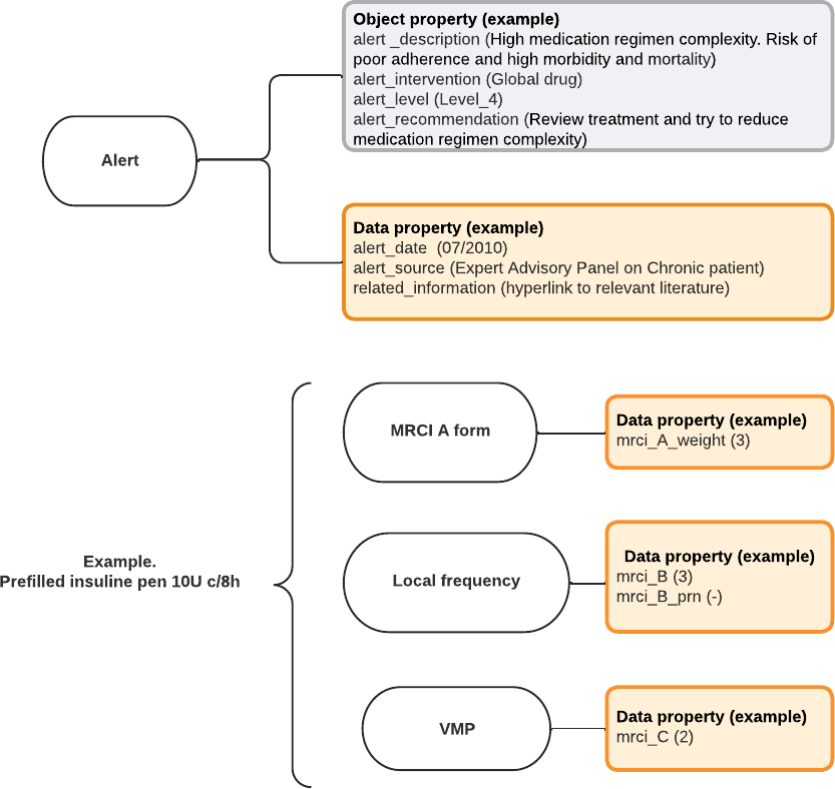
Class diagram to model medication regimen complexity. Circles represent the classes needed to quantify drug regimen complexity. Squares represent the attached properties (object or data) within each class. An example is given in brackets. MRCI: Medication Regimen Complexity Index; VMP: virtual medicinal product.

To represent the weighted scores for additional administration instructions (section C) related to taking medication with or without food, we introduced 1 attached property (“mrci C”) within the class “Virtual medicinal product (VMP)” (*Drugs* ontology). A virtual medicinal product is an abstract representation of an active medicinal ingredient associated with strength information and a route of administration (eg, “omeprazole 20mg capsule”). We assigned a total of 6257 weights. Figure 5 provides a class diagram to model medication regimen complexity, which is explained with an example.

#### Anticholinergic and Sedative Drug Burden

The OWL concept that was used to enter the data on anticholinergic and sedative drug burden was “DBI” (*DSS* ontology), in reference to the scale used for its calculation. Because the DBI is a dose‐related measure of anticholinergic and sedative drug exposure, we created the “DBI” concept as a subclass of the “Dose appropriateness” concept. To provide enough information to calculate the DBI, we introduced the property “medd,” which describes the minimum effective daily dose of each drug.

**Figure 6.**
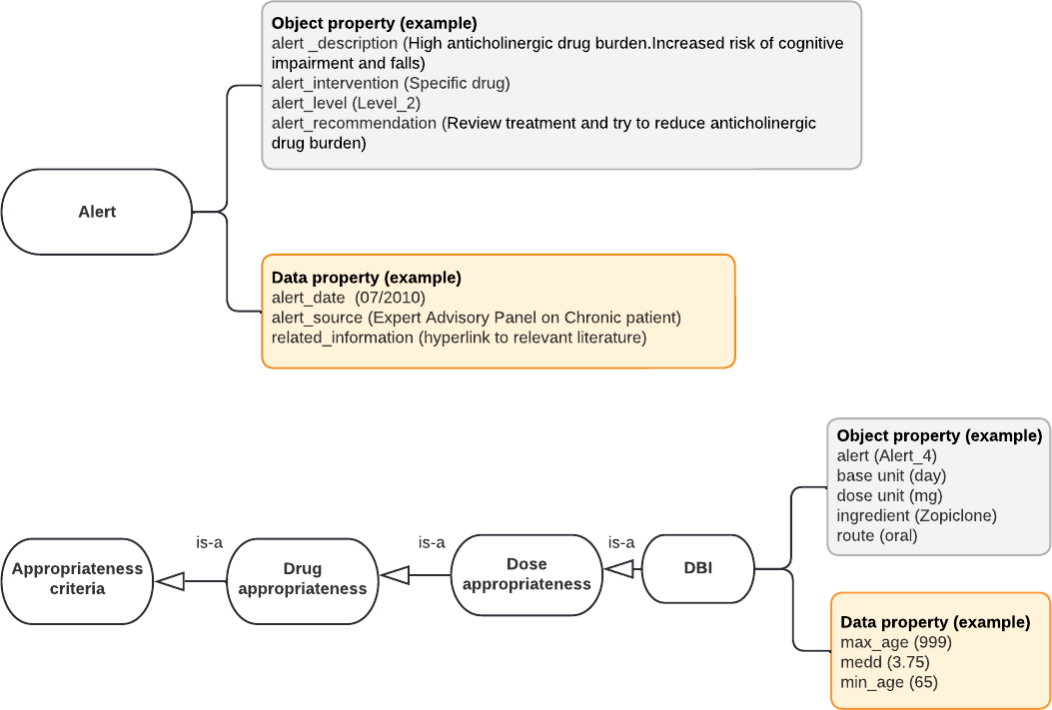
Class diagram to model anticholinergic and sedative drug burden. Circles represent the classes needed to quantify anticholinergic and sedative drug burden. Squares represent the attached properties (object or data) within each class. An example is given in brackets. DBI: Drug Burden Index.

The “DBI” class contains 164 individuals. Each individual contains the following knowledge: ingredient (eg, alprazolam), route of administration (eg, oral), age range (eg, 65-999 years), minimum effective daily dose (eg, 0.5), unit (eg, mg), base unit (eg, every 24 hours), and alert-related data. Figure 6 provides a class diagram to model anticholinergic and sedative drug burden, which is explained with an example.

#### Triggers

The OWL concept that was used to enter the triggers for detecting ADEs in older patients with multiple chronic conditions was “Trigger tool” (*DSS* ontology). We created the “Trigger tool” concept as a subclass of the “Appropriateness lab test” concept because we only included triggers based on abnormal laboratory values. Introducing new properties was not required.

**Figure 7.**
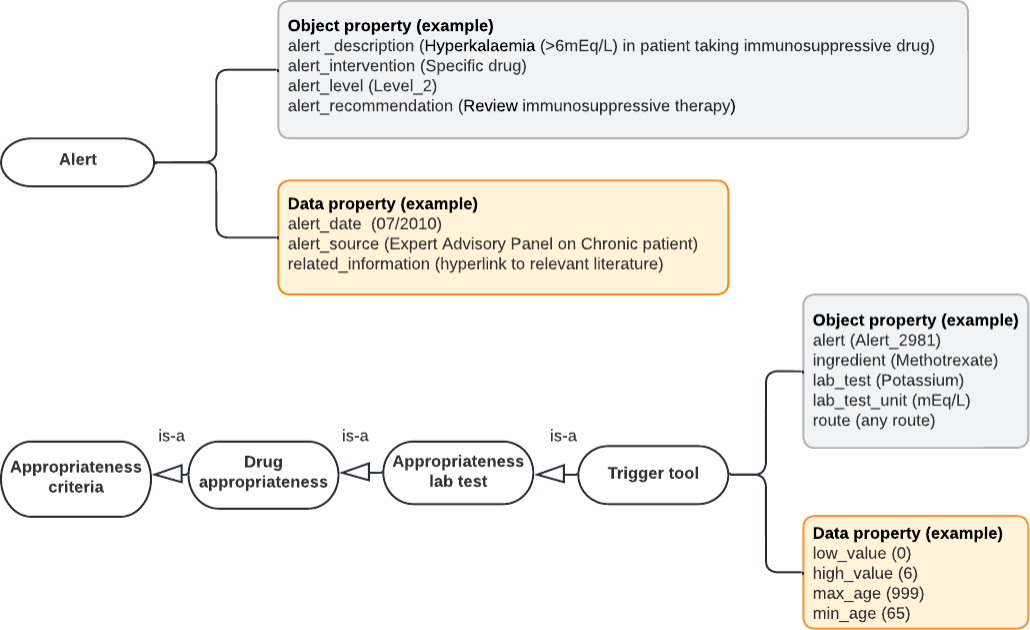
Class diagram to model triggers for detecting adverse drug events in patients with multimorbidity. Circles represent the classes needed to identify triggers of a possible adverse drug event. Squares represent the attached properties (object or data) within each class. An example is given in brackets.

The “Trigger tool” class contains 821 individuals. Each individual contains the following knowledge: ingredient (eg, furosemide), route of administration (eg, parenteral), age range (eg, 65-999 years), lab test (eg, serum glucose), lab test unit (eg, mg/dL), low value (eg, 0), high value (eg, 110), and alert-related data. Figure 7 provides a class diagram to model triggers for detecting ADEs in patients with multimorbidity, which are explained with an example.

#### Alerts

In addition to the abovementioned actions, we made changes related to the “Alert” class (*DSS* ontology). The “Alert” class included the information that was displayed when appropriateness criteria were not met. The following fields related to the “Alert” class remained unchanged: alert description, alert recommendation, alert source, alert date (the date when the alert was last updated), and related information (supporting documentation). In addition to the existing instances of the “Alert level” class (“not recommended,” “contraindicated,” or “unallowed prescription”), we created a new one called “risk minimization.” We also introduced a new class—the “Drug intervention” subclass of the “Alert” class (*DSS* ontology). We defined the following two types of drug intervention: “global drug” intervention for when a complete treatment revision is required (eg, high MRCI) and “specific drug” intervention for when a partial treatment revision is required (eg, high DBI).

### Knowledge Not Represented Using Ontologies

With regard to medication regimen complexity, we did not represent complexity based on the additional instructions related to taking multiple units at one time or needing to break or crush a tablet.

The triggers used to identify ADEs can be abnormal laboratory values, the use of certain medications or antidotes, or changes in clinical status that may indicate a possible medication-related harm. We only represented triggers based on abnormal laboratory values.

### OntoPharma-Driven Alert Module Adaptation

The OntoPharma-driven alert module works the same as it did in our previous study [22]. Once the patient-specific clinical data are sent from the CPOE system and EHR to the ontologies, local concepts are matched to their equivalent OntoPharma concepts. With regard to the chronic patient domain, we defined the following decision rules.

The MRCI is obtained by summing the scores of the three sections.

The section A score is estimated by considering the dosage form and route of administration combination. Because administering the same dosage form more than once is easier than administering different dosage forms, each dosage form and route of administration combination is counted only once within a regimen. For example, if a patient’s regimen consists of taking 3 tablets orally, their component A score is 1, not 3.

Section B and C scores are estimated by considering the dosage frequency and the virtual medicinal product prescribed, respectively. The cutoff point selected for triggering an alert (MRCI≥40) was determined by the expert advisory panel, in accordance with the literature.

The total DBI is calculated with the equation DBI=∑D/(δ+D), where “D” is the daily dose taken by the individual patient and “δ” is the minimum effective daily dose for that drug. The daily dose taken for each DBI medication is estimated by considering the dose, dose unit, and frequency. We have defined conversion factors for cases where the drug dose unit prescribed is different from the unit dose defined in the ontologies. The minimum effective daily dose is represented in the *DSS* ontology for each ingredient and route of administration combination. The cutoff point selected for triggering an alert (DBI≥1) was determined by the advisory panel, in accordance with the literature.

To evaluate the presence of triggers for identifying ADEs, we consider the ingredient regardless of the dosage. If a patient has several laboratory values, we consider the most recent values. An alert is triggered when a value is outside of the defined range [33].

Medications prescribed “as needed” were not considered in previous cases.

With regard to the interface, alerts are shown in different colors (red, orange, and yellow) according to their clinical relevance (contraindicated, moderate relevance, and low relevance). We added a new label (blue) to identify alerts aimed at risk minimization. To date, the advisory text contains the generic drug name, a short description of the possible concern, and a recommendation for improving medication appropriateness. The generic drug name is still displayed if the alert requires “specific drug” intervention. In cases where the alert requires “global drug” intervention, the text “Review total treatment” is displayed. We also included a hyperlink to relevant literature.

Alerts related to the chronic patient domain were defined, such as soft-stop alerts, so that the clinician can decide whether to ignore or accept the alert. To avoid alert fatigue, if an alert is ignored once, it will not be displayed again.

The interface that displays the alerts also includes a link to a user guide and an activity registry that serves as traceability system.

Despite the addition of new use cases, the results show that the response time for generating decision support remains short (within milliseconds), with minimal impact on the user’s workflow.

### Implementation of the Version of OntoPharma Containing the Chronic Patient Domain in a Hospital Setting

A total of 107 patients were included. The median age was 86 (IQR 80-90) years, and the majority of patients were women (n=63, 58.9%). The median length of hospital stay was 8 (IQR 5-13) days. Patients had a median of 15 (IQR 11-19) medications.

Of the 107 patients, 96 (89.7%) received at least one alert. OntoPharma generated 364 alerts (mean 3.9, SD 5.3 alerts per patient). Of these, 296 (81.3%) alerts were considered of low relevance, and 68 (18.7%) aimed at risk minimization. Further, 154 (42.3%) alerts were accepted.

Details of the types of alerts and the acceptance rates are included in Table 1. The most frequent alerts were alerts due to high anticholinergic and sedative drug burden (231/364, 63.5%), followed by alerts due to high medication regimen complexity (68/364, 18.7%) and alerts due to the presence of triggers (65/364, 17.8%).

**Table 1.** Description of the types of alerts generated by OntoPharma and the acceptance rates.

Type of alert	Frequency (N=364), n (%)	Acceptance rate, n (%^a^)
Medication regimen complexity	68 (18.7)	40 (58.8)
Anticholinergic and sedative drug burden	231 (63.5)	84 (36.4)
Triggers	65 (17.8)	30 (46.2)

a Percentages were calculated by using the numbers in the “Frequency” column as denominators.

## Discussion

### Principal Results

This paper presents a modeling approach, which was formalized in ontological terms, for defining the chronic patient domain that provides support for improving medication appropriateness in older patients with multimorbidity. The chronic patient domain was built on OntoPharma—an ontology-based CDSS for reducing medication prescribing errors that has already been implemented in a tertiary referral hospital [22].

There are already ontology-based CDSSs that address medication management in patients with chronic conditions [37]. However, to the best of our knowledge, this is the first ontology-based approach that models medication regimen complexity, anticholinergic and sedative drug burden, and triggers for identifying possible adverse events. Farrish and Grando [38] built an ontology to assist with the management of polypharmacy prescriptions for patients with multiple chronic conditions to reduce the overall treatment complexity. Recently, Román-Villarán et al [39] developed an ontology-based CDSS for patients with complex chronic conditions. However, the knowledge sources used were different from ours, including clinical practice guidelines, the LESS-CHRON (List of Evidence-Based Deprescribing for Chronic Patients) criteria, and the STOPP/START (Screening Tool of Older Persons’ Prescriptions and Screening Tool to Alert to Right Treatment) criteria, among others. These ontology approaches for patients with chronic conditions have been validated with patient data from databases. However, it is important to note that they are not implemented in a real environment, unlike our ontology approach. OntoPharma provides rapid and real-time support to improve medication appropriateness in older patients with multimorbidity.

Using ontologies instead of relational databases, which are the predominant choice in current commercial CDSSs, has distinct advantages [40,41]. First, the semantic approach and the use of OWL enable a convenient infrastructure for reuse. Hence, we reused the existing OntoPharma framework, without having to start from scratch. In addition, ontologies are more flexible and efficient in dealing with changes; thus, it was possible to add a new domain to OntoPharma without major complications. We were able to model a complex domain, creating only 6 new classes and 5 new properties. This was possible because the three ontologies (*Drugs*, *DSS*, and *Local pharmacy*) are interconnected (Figure 1), and classes are linked between them through object properties.

To ensure flexibility, scalability, and sustainability, we operated on the most appropriate level of abstraction. To define anticholinergic drug burden and triggers, we considered the ingredient. However, to define weighted scores for additional administration instructions (MRCI section C), we considered the class “Virtual medicinal product (VMP).”

To integrate structured clinical data with clinical knowledge, we reused the mappings previously established in the ontology *Local pharmacy*. It was only necessary to add some new mappings related to laboratory parameters.

End users participated throughout the development of the chronic patient domain in order to ensure usability and gain user acceptance [42,43]. As a result, we introduced some changes in the user interface, such as new clinical relevance levels and a hyperlink to relevant literature. Some proposals for improvement, such as showing the laboratory values next to the alert, have not been implemented yet. Usability may also be influenced by the response time for generating decision support. However, response time has not been modified, showing that OntoPharma is scalable.

### Limitations

In terms of evaluation, we have not identified a database-based system for direct comparison with OntoPharma. van der Sijs et al [44] conducted a systematic review, concluding that drug safety alerts are overridden by clinicians in 49% to 96% of cases. Our acceptance rate (154/364, 42.3%) was expected to be better, considering that an expert advisory panel selected the most useful information to improve medication appropriateness in older patients. This may be partly explained by the following limitations. First, appropriateness criteria were evaluated if patients were older than 65 years. Considering that the older population is heterogeneous, we should also have considered frailty—a known factor indicative of vulnerability to medication-related problems [45]. Second, the alerts with a lower acceptance rate (84/231, 36.4%) were related to the DBI. Interventions for reducing the DBI commonly involve progressive medication deprescribing, which is difficult to realize in a tertiary hospital and would be easier in intermediate care [46]. On the other hand, poor adherence is one of the major consequences of high MRCI scores [23]. In hospitals, the administration of medications is primarily the nurses’ responsibility; therefore, clinicians may have not given sufficient importance to MRCI alerts. Acceptance rates might improve in outpatient care. Our research focused primarily on clinician decision-making. The variables analyzed allowed us to identify the scale of potentially inappropriate medications and the usefulness of OntoPharma. However, evaluating OntoPharma’s influence on health outcomes is a challenge that we should take up in future.

As mentioned in our previous paper on OntoPharma [22], one limitation of this study is maintaining the evidence and keeping it relevant and up to date [47]. To create individual instances, we extracted the information from papers. Since this was not done via automatic extraction, it was a time-consuming process. With regard to the maintenance, we must assign a complexity weight if there is a new dose form or dosing frequency; these data are not updated frequently. In addition, we must check if new medications have additional administration instructions, are capable of causing anticholinergic adverse effects, or are included in the set of triggers for detecting ADEs in older patients.

Of note, although mapping in this study did not take excessive time, we are aware that manual mapping is a resource-intensive and ongoing process.

We have not represented all of the knowledge from the sources of information. OntoPharma relies on structured data; therefore, we have prioritized representing data that are structured in text format within the EHR. As a result, medication regimen complexity may be underestimated because special instructions are underrepresented. In addition, there are triggers that are different from abnormal laboratory values that are not represented in ontologies. Even though there exist large amounts of health care data, the main challenge to improving results of CDSSs is converting free-text data into structured fields computationally [48].

In future implementations, we will continue to represent complex drug knowledge that is absent in commercial databases. We are currently modeling knowledge for supporting the neonatal population and populations at risk for hepatitis B virus reactivation. To capture clinicians’ reasoning processes, we must place a high priority on increasing structured patient data within EHRs.

Other areas for future work are mentioned in our previous OntoPharma paper [22], such as developing a more complex CDSS that can be applied across the entire treatment process and is not only restricted to the medication prescription process. Finally, we must continue working on customized alerts to avoid alert fatigue [49].

Despite the limitations, we believe that our methods have been successful in modeling knowledge related to the chronic patient domain and that the proposed version of OntoPharma is an enhancement of the previous one. Although optimizing care in older patients is a context-dependent, complex process, we believe that developing an ontology to support the chronic patient domain constitutes a major step toward improving medication appropriateness in a generalizable and reusable way.

### Conclusions

Polypharmacy in the older population poses challenges to the delivery of medical care because of the increased difficulties in guaranteeing appropriate prescription. We proposed an ontology-based approach to provide support for improving medication appropriateness in older patients with multimorbidity in a scalable, sustainable, and reusable way. OntoPharma has been implemented in clinical practice and generates alerts, considering the following use cases: medication regimen complexity, anticholinergic and sedative drug burden, and the presence of triggers for identifying possible adverse events.

## Supplementary material

10.2196/45850 Multimedia Appendix 1 Medication knowledge concepts that are represented in OntoPharma to define the chronic patient domain.

10.2196/45850 Multimedia Appendix 2 Properties and their facets, which are represented in OntoPharma to define the chronic patient domain.

## Abbreviations

ADE: adverse drug event
C3RG: Central Catalonia Chronicity Research Group
CDSS: clinical decision support system
CPOE: computerized physician order entry
DBI: Drug Burden Index
DSS: Decision support system
EHR: electronic health record
LESS-CHRON: List of Evidence-Based Deprescribing for Chronic Patients
MRCI: Medication Regimen Complexity Index
MRCI-E: Spanish Medication Regimen Complexity Index
OWL: Web Ontology Language
STOPP/START: Screening Tool of Older Persons' Prescriptions and Screening Tool to Alert to Right Treatment
