# Supplementary material for: An Ontology-Based Approach to Improving Medication Appropriateness in Older Patients: Algorithm Development and Validation Study
Source: JMIR Med Inform. 2023 Jul 10;11:e45850. doi: 10.2196/45850 (PMC10366962; doi:10.2196/45850)
Supplement: Multimedia Appendix 1 [file medinform-v11-e45850-s001.docx]

| **Supplementary file 1: Medication knowledge concepts represented in OntoPharma to define chronic patient domain*** | |
| --- | --- |
| **Drugs ontology** | |
| **Concept** | **Concept definition** |
| Active ingredient | Any substance or combination of substances used in a pharmaceutical product, intended to furnish pharmacological activity or to otherwise have direct effect in the diagnosis, cure, mitigation, treatment or prevention of disease. |
| AI base | Active ingredient as the core or base molecule |
| AI derivative | The different salts, esters, ethers, isomers, mixtures of isomers, complexes or derivatives of an active ingredient |
| Composition unit | Minimum unit in which the dose is administered |
| Drug | A medicine or other substance which has a physiological effect when ingested or otherwise introduced into the body |
| Drug dose form | The physical form of a dose of a drug |
| Drug route | Route of administration for drugs |
| Matter | [Material](https://dictionary.cambridge.org/es-LA/dictionary/english/material) with [particular](https://dictionary.cambridge.org/es-LA/dictionary/english/particular) [physical](https://dictionary.cambridge.org/es-LA/dictionary/english/physical) [characteristics](https://dictionary.cambridge.org/es-LA/dictionary/english/characteristic) |
| Medicinal product | A medicine or other substance which has a physiological effect when ingested or otherwise introduced into the body |
| Presentation unit | Unit of measurement containing the dose of active ingredient |
| Product ingredient | Any substance or combination of substances used in a pharmaceutical product, intended to furnish pharmacological activity or to otherwise have direct effect in the diagnosis, cure, mitigation, treatment or prevention of disease. |
| Strength unit | Unit of measurement for the active ingredient quantity |
| Units | Units of measure |
| Unit equivalence | Conversions between units |
| VMP | A virtual therapeutic moiety is an abstract representation of an active medicinal ingredient or substance devoid of strength and form, which when formulated as a medicinal product, is intended for use in preventing or treating diseases in patients |
| VPI | A virtual product ingredient is an ingredient used in a virtual medicinal product |
| **DSS ontology** | |
| **Concept** | **Concept definition** |
| Alert | Types of alerts |
| Alert description | Text describing the causes of medication errors |
| Alert level | Alert levels according to the clinical relevance |
| Alert recommendation | Text describing the recommendation to avoid a medication error |
| Appropriateness criteria | [Prescribing](https://www.lawinsider.com/dictionary/therapeutic-appropriateness) criteria based on scientific evidence |
| Appropriateness lab test | Drug appropriateness criteria based on a laboratory test |
| DBI | Drug appropriateness criteria based on anticholinergic and/or sedative drug burden |
| Dose appropriateness | Drug appropriateness criteria based on dose adjustment |
| Drug appropriateness | [Drug prescribing](https://www.lawinsider.com/dictionary/therapeutic-appropriateness) criteria based on scientific evidence |
| Drug intervention | Type of alert when a drug intervention is required |
| Global drug | Type of alert when complete treatment revision is required |
| Lab test | Types of laboratory test |
| Multilingual text | Text shown in the alerts |
| MRCI A form | Dosage form/route of administration combination |
| Specific drug | Type of alert when partial treatment revision is required |
| Trigger tool | Drug appropriateness criteria based on the presence of triggers |
| **Local pharmacy ontology** | |
| **Concept** | **Concept definition** |
| Local concept | Concepts used in the local environment |
| Local drug | Drugs used in the local environment |
| Local form | Types of dosage forms used in the local environment |
| Local frequency | Frequencies (how often the medication is to be administered as events per unit of time) used in the local environment |
| Local lab test | Laboratory tests used in the local environment |
| Local route | Route of administration for drugs used in the local environment |
| Local unit | Units of measure used in the local environment |
| *New concepts are highlighted in yellow | |
