# Supplementary material for: An Ontology-Based Approach to Improving Medication Appropriateness in Older Patients: Algorithm Development and Validation Study
Source: JMIR Med Inform. 2023 Jul 10;11:e45850. doi: 10.2196/45850 (PMC10366962; doi:10.2196/45850)
Supplement: Multimedia Appendix 2 [file medinform-v11-e45850-s002.docx]

| **Supplementary file 2: Properties and their facets represented in OntoPharma to define chronic patient domain*** | | | |
| --- | --- | --- | --- |
| **Drugs** | | | |
| **Domain** | **Property** | **Range** | **Object (O) or Datatype (D)** |
| Product_Ingredient | active_ingredient | Active_ingredient | O |
|  | comp_unit | Unit | O |
|  | presentation_unit | Unit | O |
|  | strength_unit | Unit | O |
|  | strength_comp | Float | D |
|  | strength_presentation | Float | D |
| VMP | dose_form | Drug_dose_form_type | O |
|  | drug_route | Drug_route_type | O |
|  | main_route | Drug_route_type | O |
|  | multi-dose | Boolean | D |
|  | multi-ingredient | Boolean | D |
|  | mrci C | Float | D |
|  | Vpi | Product_Ingredient | O |
| **DSS** | | | |
| **Domain** | **Property** | **Range** | **Object or Datatype** |
| Alert | alert_date | String | D |
|  | alert_description | Alert_description | O |
|  | related_information | anyURI | D |
|  | alert_level | Alert_level | O |
|  | alert_recommendation | Alert_recommendation | O |
|  | alert_source | String | D |
| Alert_level | level | int | D |
| Appropriateness_criteria | alert | Alert | O |
|  | max_age | Float | D |
|  | min_age | Float | D |
| Appropriateness_lab_test | lab_test | Lab_test_type | O |
|  | Lab_test_unit | dmm:Unit | O |
|  | high_value | Float | D |
|  | low_value | Float | D |
| DBI | medd | Float | D |
| Dose_appropriateness | base_unit | dmm:Unit | O |
|  | dose_unit | dmm:Unit | O |
| Drug_appropriateness | ingredient | dmm:Ingredient | O |
|  | route | dmm:Drug_route_type | O |
| MRCI A form | mrci A weight | int | D |
| **Local_Pharmacy** | | | |
| **Domain** | **Property** | **Range** | **Object or Datatype** |
| Local_concept | local_code | String | D |
|  | ontopharma_concept | dmm:Ontopharma_concept | O |
| Local_frequency | daily_frequency | Float | D |
|  | mrci B | Float | D |
|  | mrci B PRN | Float | D |
| Local_lab test type | validity_period | Float | D |
|  | validity_period_unit | dmm:Unit | O |
| *New properties are highlighted in yellow | | | |
